# Cross-Reactivity between *Schistosoma mansoni* Antigens and the Latex Allergen Hev b 7: Putative Implication of Cross-Reactive Carbohydrate Determinants (CCDs)

**DOI:** 10.1371/journal.pone.0159542

**Published:** 2016-07-28

**Authors:** Michael J. Doenhoff, Marwa El-Faham, Susan Liddell, Heidi R. Fuller, Ronald G. Stanley, Gabriele Schramm, Joseph E. Igetei

**Affiliations:** 1 School of Life Sciences, University of Nottingham, University Park, Nottingham, NG7 2RD, United Kingdom; 2 School of Biosciences, Sutton Bonington Campus, University of Nottingham, Nottingham, LE12 5RD, United Kingdom; 3 School of Biological Sciences, University of Wales, Bangor, Gwynedd, LL57 2UW, United Kingdom; 4 Research Center Borstel, Priority Area Asthma and Allergy, Experimental Pneumology, Parkallee 22, D-23845, Borstel, Germany; 5 Department of Medical Parasitology, Faculty of Medicine, Alexandria University, Alexandria, Egypt; 6 Department of Animal and Environmental Biology, Faculty of Life Sciences, University of Benin, Benin City, Edo State, Nigeria; Instituto Butantan, BRAZIL

## Abstract

IgG antibodies produced by rabbits immunized against *S*. *mansoni* antigens cross-reacted with aqueous soluble constituents of a variety of allergens. The antibody cross-reactivity was largely sensitive to degradation by treatment of the target antigens with sodium meta-periodate, suggesting the cross-reactivity was due to carbohydrate determinants that were common to both the schistosome and the allergens (CCDs). The reaction between the rabbit antibodies and a 43 kDa molecule in a rubber latex extract was analysed further: tandem mass spectrometry identified the latex molecule as allergen Hev b 7. Rabbit anti-schistosome IgG antibodies purified by acid-elution from solid-phase latex Hev b 7 reacted with the *S*. *mansoni* egg antigens IPSE/alpha-1 and kappa-5 and cercarial antigens SPO-1 and a fatty acid-binding protein. Moreover, purified anti-*S*. *mansoni* egg, latex cross-reactive antibodies reacted with antigenic constituents of some fruits, a result of potential relevance to the latex-fruit syndrome of allergic reactions. We propose that IgG anti-schistosome antibodies that cross-react with allergens may be able to block IgE-induced allergic reactions and thus provide a possible explanation for the hygiene hypothesis.

## Introduction

In areas with advanced health systems there has in recent decades been a steep rise in the incidence of asthma, allergies and other disorders of the human immune system [1]. The ‘hygiene hypothesis’ is often invoked as an explanation, i.e., due to more hygienic living conditions and/or their prevention by vaccinations, populations have become less afflicted by parasitic and microbial infections (sometimes referred to as ‘old friends’ [2]) and the patterns of maturation of human immune systems are therefore now different from those that pertained to pre-hygienic, infection-rife eras [3]. One consequence of this dysfunction is an inappropriate and pathological immune response to environmental and air-borne antigens that are the causes of asthma and allergies, and which ‘normal’ immune responses would have rendered innocuous. The hypothesis may also apply to autoimmune and inflammatory diseases such as Type 1 diabetes and inflammatory bowel disease [4, 5].

Immunoglobulin E (IgE) antibody plays a fundamental role in the pathogenesis of allergy [6] and asthma [7]. IgE binds with high affinity to its Fc receptors (FcεRI) on tissue mast cells or blood basophils and when an allergen molecule reacts with specific IgE antibody on the surface of one of these cells it is triggered to release mediators of inflammation such as histamine and prostaglandins. The IgE is produced by B lymphocytes which are under the control of cytokines such as interleukins 4, 5 and 13 (IL-4, IL-5, IL-13) that are produced by Th2 cells. These are a subpopulation of T-helper (Th) lymphocytes distinguished from Th1 cells, a principal cytokine product of which is interferon gamma (IFNγ) [8]. Allergies and asthma are the outcome of a disordered immune response in which Th2 cells are the main driving force and the production of specific IgE antibody results in a propensity for hypersensitive reactivity against allergen molecules.

It is generally acknowledged that Th2 cell-driven immune responses evolved to give immunological protection against macro-parasitic (helminth) infection [9–13], with involvement in tissue repair and wound healing another possible attribute [14]. Different parasites may, however, be susceptible to only a selection of the wide range of different immune effector mechanisms generated by a Th2 response. In some instances, as a result of ‘immune evasion’ by the parasite and/or its capacity to modulate the immune responses generated against it (‘immunomodulation’ or ‘modified Th2 responsiveness’), the host fails to clear a helminth infection, which thus becomes chronic.

Modulation of immune responsiveness can be driven by regulatory T cells [15], B cells [16] and/or alternatively-activated or M2 macrophages [17]. Regulatory T cells (Tregs) are considered essential for maintaining peripheral tolerance, preventing autoimmune diseases and limiting chronic inflammatory disease [18]. A common explanation for the hygiene hypothesis, i.e., the relative absence of allergies and other immune disorders in those infected with parasites, invokes the actions of Tregs and their cytokine products [19–21] or Bregs [22, 23].

So-called ‘blocking antibodies’ provide another possible explanation for the hygiene hypothesis. Three types of blocking antibodies have been proposed: firstly, high concentrations of specific and non-specific IgE, as often induced by helminth infections, may occupy Fcε receptors on mast cells and prevent access to them by allergen-specific IgE. Good evidence to support this possibility and others invoking IgE-dependent blocking activity has not, however, been found [24, 25] as has been reviewed elsewhere [19].

Secondly, IgG antibodies, including IgG4, are also produced during chronic helminth infections [26–28]. IgG4 production is driven by IL-10 [29]. There are similarities between immunomodulated chronic helminth infections and the outcome of successful immunotherapy for allergies as the latter are characterized by high IgG4:IgE ratios, Treg activity [30, 31], high levels of IL-10 [32] and a lack of clinical allergic reactivity (despite the presence of allergen-specific IgE).

Thirdly, antibodies that react with carbohydrate epitopes that are common to both helminths and allergens, so-called cross-reactive carbohydrate determinants (CCDs) (Altmann, 2007), may have ‘blocking’ activity.

In preliminary experiments we found that rabbit IgG antibodies raised against Sm480, a high molecular weight, glycosylated *Schistosoma mansoni* antigen [33], cross-reacted extensively with a great variety of the constituents of plants and invertebrates that are associated with allergic reactions in humans. Rabbit anti-Sm480 antibodies had previously been shown to react strongly against fucose-containing epitopes of schistosome glycolipids [34, 35] and the cross-reactivity of the anti-Sm480 antibodies with allergens could be abolished by treatment of the membranes carrying the electroblotted allergen molecules with a solution of sodium meta-periodate, a procedure which destroys the integrity of pentoses and hexoses by oxidation [36]. The antigenic cross-reactivity between Sm480 and molecules in allergen extracts might therefore be due to CCDs [37]. The existence of carbohydrate structures common to plants and invertebrates, giving rise to the possibility of antigenic cross-reactivity, has been known for many years [38–43]. The concept has included epitopes possessed by helminth parasites [44].

In this study IgG antibodies from rabbits immunized with unfractionated extracts of *S*. *mansoni* eggs were found to react with allergen extracts similarly to the anti-Sm480 antibodies. The polyspecific anti-*S*. *mansoni* antisera were then used for further study of the schistosome/allergen antigenic cross-reactions. Initially, what might be called ‘proof-of-principle’ investigations were centred on a 43 kDa constituent of natural rubber (*Hevea brasiliensis*) latex which was one of only a few molecules reacted against in western blots by the anti-Sm480 and the anti-*S*. *mansoni* polyspecific sera, in contrast to the multiple cross-reactivities given by these sera against extracts of most of the other allergens.

We purified the schistosome cross-reactive latex molecule and determined its identity by tandem mass spectrometry. We also found that schistosome egg and cercarial antigens (other than Sm480) seemingly have epitopes that are also present on the latex molecule and thus are potentially responsible for the schistosome/latex antigenic cross-reactivity.

Interestingly, we also observed that anti-schistosome IgG that reacted with the 43 kDa latex allergen reacted also with antigenic constituents of different foods, suggesting relevance of this study to the so-called latex-fruit syndrome [45–47]. We therefore investigated to what extent anti-schistosome antibodies that reacted with the 43 kDa latex molecule reacted also with antigenic constituents of different foods.

We discuss the possibility that IgG anti-schistosome antibodies that cross-react with allergens may be able to block IgE-induced allergic reactions and thus provide a possible explanation for the hygiene hypothesis.

## Materials and Methods

### *S*. *mansoni* life cycle and preparation of aqueous extracts of *S. mansoni* antigens

Experiments that used mice used for production of *S*. *mansoni* antigens were approved by the Ethical Review Committee of the university in which these materials were produced and the work was carried out in strict accordance with UK government regulations for animal welfare and amelioration of suffering in force at the time. The work was licensed under legislation specified by the UK Animals (Scientific Procedures) Act 1986, (project licence numbers PPL 40/3024 and 40/3595). Animals were killed by administration of a lethal dose of pentobarbitone anaesthetic.

The life cycle of a Puerto Rican isolate of *S*. *mansoni* was maintained by passage through *Biomphalaria glabrata* snails and random-bred laboratory mice as described previously [48].

Cercariae were obtained by light-induced shedding from snails with patent infections and the concentrated larvae processed for extraction of soluble antigens (SmCH) as described [49]. *S*. *mansoni* cercarial transformation fluid (SmCTF), a solution containing soluble forms of cercarial glycocalyx generated from schistosomula after mechanical transformation of cercariae, was prepared as described previously [50]. Adult *S*. *mansoni* worms were obtained by perfusion from incised hepatic portal veins of lethally-anaesthetized mice that had been given a percutaneous infection of 200 cercariae 42 days previously, as described by [51] and extracts containing soluble adult worm antigens (SmWH) were prepared as described [49]. *S*. *mansoni* eggs were isolated from the livers of mice with patent infections as described [52] and aqueous extracts containing soluble egg antigens (SmSEA) were prepared from the eggs as described [53].

### Preparation of extracts of allergens

An extract of rubber latex was prepared from Copydex, a commercial product made from natural rubber-tree (*Hevea brasiliensis*) latex sap and sold in the UK as a carpet adhesive (Henkel, Winsford, Cheshire, UK CW7 3QF). 10 ml of Copydex was diluted with 10 ml of isotonic phosphate-buffered saline, pH 7.4 (PBS) and gently mixed for 30 minutes. The homogenate was sonicated three times at 5,000 Hz for 20 seconds each time with 20 second intervals and then centrifuged at 14,000 x g for 30 minutes at 4°C. A relatively transparent aqueous phase near the base of the centrifuged mixture was removed and dispensed in 100 μl aliquots into 0.5 ml micro-centrifuge tubes and stored at -20°C until required for use.

Ripe bananas (*Musa* spp), strawberries (*Fragraria ananassa*), ripe melon (*Cucumis melo*), avocado (*Persea americana*), tomatoes (*Lycopersicon esculatum*), kiwi fruit (*Actinidia deliciosa*), cultivated mushrooms (*Agaricus* spp.), and prawns (*Pandalus borealis*) were bought from local groceries and approximately 20 g of each were separately ground into a fine paste in a ceramic pestle and mortar. Each homogenate was collected in a 50 ml centrifuge tube, diluted with an equal volume of PBS. (Because of the amount of fluid present after homogenization, the melon extract required no further dilution after homogenization.) The homogenates were agitated gently for 30 minutes at room temperature after which they were centrifuged at 14,000 x g for 10 minutes at room temperature. The individual supernatants were divided into 100 μl aliquots and stored at -20°C. Bodies of adult cockroaches (*Blatta orientalis*) were treated similarly except that the initial quantity was 5 g and the volume of PBS reduced accordingly.

To extract peanuts (*Arachis hypogaea*) and edible chestnuts (*Castanea sativa*) approximately 20 g uncooked nuts bought from a local grocery were crushed and ground as finely as possible using a pestle and mortar. Larger particles were removed by sifting through a metal sieve of 180 μm mesh size. The sieved material was placed in a 50 ml centrifuge tube and diluted with an equal volume of PBS. The mixture was sonicated 3 times, with 20 second bursts each time at 20 second intervals and centrifuged at 10,000 x g for 10 minutes at room temperature. The resultant supernatants containing water-soluble nut proteins were removed, divided into 100 μl aliquots and stored at -20°C until required for use.

Plant pollens: 50 mg of pollen from Timothy grass (*Phleum pratense*), ragweed (*Ambrosia* spp.) and birch tree (*Betula verrucosa*) were placed separately in 1 ml 0.05 M Tris-HCl buffer, pH 7.5, and extracted as described previously [54]. The resulting supernatants were divided into 100 μl aliquots and stored at -20°C until use.

Bee venom and house dust mite: 10 mg bee (*Apis mellifera*) venom (Sigma, Poole, Dorset) or 40 mg house dust mite (*Dermatophagoides farinae* powder, kindly donated by Dr Beverly Lees of Allergy Therapeutics, Worthing, Sussex, UK) were separately added to 1 ml volumes of PBS and the mixtures agitated gently for 30 minutes at room temperature. The suspensions were centrifuged at 10,000 x g for 10 minutes at room temperature, the supernatants removed, divided into 100 μl aliquots and stored at -20°C.

### Protein concentrations

The protein concentration in SmSEA and in each allergen extract was estimated using the BioRad DC Protein Assay (BioRad Laboratories, Inc., Hercules, California USA), using bovine serum albumin as the reference standard.

### Preparation of polyspecific and monospecific antisera against *S*. *mansoni* antigens and allergens

Work to produce rabbit antibodies was approved by the University’s Ethical Review Committee and carried out in strict accordance with UK government regulations for animal welfare and amelioration of suffering in force at the time (2001–2002). The project was licensed under legislation specified by the UK Animals (Scientific Procedures) Act 1986, (licence no. PPL 40/2278). Animals were killed by administration of a lethal dose of pentobarbitone anaesthetic.

Polyspecific rabbit antisera were raised against *S*. *mansoni* eggs or cercariae. The respective schistosome antigen solutions were emulsified in complete Freund’s adjuvant as described [49, 53]. Injections of total volumes of 1 ml, which contained approximately 5 mg protein, were administered to rabbits in 0.1 ml quantities both intramuscularly (in both hind legs) and subcutaneously with 0.1 ml of the antigenic material at multiple dorsal sites once a week to a total of 1 ml. Injections were continued until a strong antibody response was obtained against the immunogen when the antisera were tested in immunoelectrophoresis. Rabbits were exsanguinated by cardiac puncture and sera were collected and stored at -20°C until required. Sera from rabbits injected with Freund’s adjuvant alone, developed as scheduled above, were used as control.

Rabbit antisera specific for four egg antigens: IPSE/ alpha-1, omega-1, kappa-5 and Sm480 were prepared as described [33, 53].

For production of rabbit anti-bee venom and rabbit anti-birch tree pollen antisera, 10 mg lyophilized *Apis mellifera* bee venom or 10 mg birch tree (*Betula verrucosa*) pollen were respectively added to 2 ml PBS. The solution/suspensions were emulsified with an equal volume of Freund’s adjuvant and respective rabbits immunized as described previously for other immunogens [53]. Both venom and pollen were kindly donated by Dr Beverly Lees of Allergy Therapeutics (Worthing, Sussex, UK).

### Sodium dodecyl sulphate polyacrylamide gel electrophoresis (SDS-PAGE), western immunoblotting and periodate treatment

One-dimensional SDS-PAGE in 12% acrylamide gels was performed as described [55], modified as in [56] in a BioRad Minigel system (Bio-Rad Laboratories, Inc., Hercules, California USA).

Western immunoblotting was done as described [57] adapted as in [53]. Rabbit antisera used as the sources of primary antibodies were diluted 1:200. Peroxidase-conjugated goat anti-rabbit IgG secondary antibodies (Sigma, Poole, Dorset, UK) were used diluted 1:1000. Membranes were incubated with the antibodies for two hours at room temperature with gentle shaking.

In order to destroy carbohydrate determinants, with minimal alteration of protein or lipid epitopes, nitrocellulose sheets carrying electroblotted antigens were incubated for 1 hour in either 10 mM or 20 mM sodium meta-periodate dissolved in 0.05 M sodium acetate buffer, pH 4.5, prior to application of primary antibody as described [36, 58, 59]. Controls were treated in the sodium acetate buffer under similar conditions, but without the meta-periodate.

### Silver-staining of SDS-PAGE gels for protein visualization

Silver staining technique for the visualisation of electrophoresed proteins in SDS-PAGE gel was done as described previously [60].

SDS-PAGE gels carrying proteins for mass spectrometric analysis were stained using SimplyBlue SafeStain (Invitrogen, Carlsbad, CA).

### Purification of a 43 kDa latex molecule for mass spectrometry

Purification of a 43 kDa latex antigen that was cross-reactive with rabbit anti-*S*. *mansoni* antibodies was achieved in one-dimensional sodium dodecyl 12% acrylamide gel electrophoresis [55] as modified as in [56]. 20 μl of latex extract were loaded into separate gels with broad wells (6.2 cm long) and electrophoresed. The stained protein bands were excised from thin strips of the polyacrylamide gels, placed in a 1.5 ml Eppendorf vial and covered with a minimum volume of elution buffer (0.06 M Tris-HCl, 10% SDS, pH 7.0) [61]. The sample was incubated at 37°C for 24 hours, centrifuged at 14,000 x g for 30 minutes at room temperature and the resultant eluate was removed. Further purification of the protein was achieved by re-electrophoresing the eluate in a fresh SDS-PAGE gel. The sequence of (i) SDS-PAGE, (ii) elution of the protein from gel strips, and (iii) re-electrophoresing in SDS-PAGE, was repeated two times in an effort to obtain a sufficiently pure sample of the protein suitable for analysis by mass spectrometry (MS).

### Mass spectrometric analysis of purified protein

Analyses of protein bands purified from gels were carried out using tandem mass spectrometry on a Waters Corporation Q-TOF 2 instrument [62, 63]. Purified protein was digested by trypsin overnight at 37°C and resultant peptides analysed using LC-MSMS. Data were searched using the MASCOT software Version 2.3.01 for peptide matching and protein identification. Amino acid sequence searches used the protein basic local alignment search tool (pBLAST) at the National Centre for Biotechnology Information (NCBI) against the non-redundant (nr) protein sequences database (http://blast.ncbi.nlm.nih.gov/Blast.cgi) to identify homologous proteins, while protein sequence alignments were produced using pairwise sequence alignment software (http://www.ebi.ac.uk/Tools/psa/psa/emboss_needle/).

### Prediction of glycans on identified proteins

The prediction of potential glycosylation sites on the amino acid sequences of proteins was done using GlycoEP software (www.imtech.res.in/raghava/glycoep/) [64]. Each amino acid sequence was uploaded or pasted onto the software, followed by ticking the option for Binary Profile of Patterns (BPP), while leaving other settings as default, and set to run, thereby revealing the number(s) and positions of potential N-linked or O-linked glycosylation site(s) on the amino acid sequence of the allergen/antigen.

### Purification of rabbit anti-*S*. *mansoni* antibodies that cross-react with the latex allergen

Rabbit antibodies that had been applied to immunoblots as the primary antibody were eluted from nitrocellulose paper (NCP) strips bearing the target antigen by incubation of the strips in low pH buffer, a method adapted from [65]. Thus, the position of the immune-complex was initially ascertained by cutting 1 cm parallel strips from each edge of the NCP and completing the western blot reaction on those ‘edge’ strips by incubation with secondary antibodies and chromogenic staining. After development and washing the two stained edges were realigned against the sides of the main part of the NCP to determine the position of the antigen and adherent primary antibodies. A horizontal strip containing the immune-complex was cut from the NCP and the rabbit antibodies eluted by placing the strip in 1 ml of 0.1 M glycine buffer, pH 2.8, and gently agitating it at room temperature for 10 minutes. The eluting buffer was removed and rapidly neutralised using 1 M Tris, pH 8.0, and the solution containing the eluted antibodies stored at 4°C. After washing the strip three times in PBS, each for 5 minutes on a rocker, it was re-incubated in the same primary antiserum for at least 2 hours at room temperature or overnight at 4°C. The process of incubation and antibody elution was repeated 4 times. Solutions of eluted antibodies were pooled and concentrated to 5% of the starting volume using Amicon ultra centrifugal filters, 3000 molecular weight cut-off, (Millipore, Carrigtwohill, Co. Cork, Ireland) and the final solution stored at 4°C.

## Results

### IgG antibodies from rabbits immunized with *S*. *mansoni* antigens cross-react with a variety of plant allergens and vice versa

Initial experiments in which antigenic cross-reactivity was observed between *S*. *mansoni* and a variety of allergens were performed with sera from rabbits immunized with Sm480, a high molecular weight, glycosylated *S*. *mansoni* egg antigen [33]. IgG antibodies in these sera cross-reacted with the constituents of aqueous extracts of a wide variety of plants and invertebrates known to be allergic in humans (S1 Fig). *Vice versa*, rabbit sera raised against two of the allergen extracts chosen at random, bee venom and birch tree pollen, reacted against a continuum of electroblotted SmSEA antigens that were mostly >30 kDa (S2 Fig). Rabbit anti-Sm480 antibodies were reactive against some antigens in SmSEA with a wide range of sizes, while serum from a rabbit injected with Freund’s complete adjuvant alone as a negative control showed no reactivity (S2 Fig).

**Fig 1:**
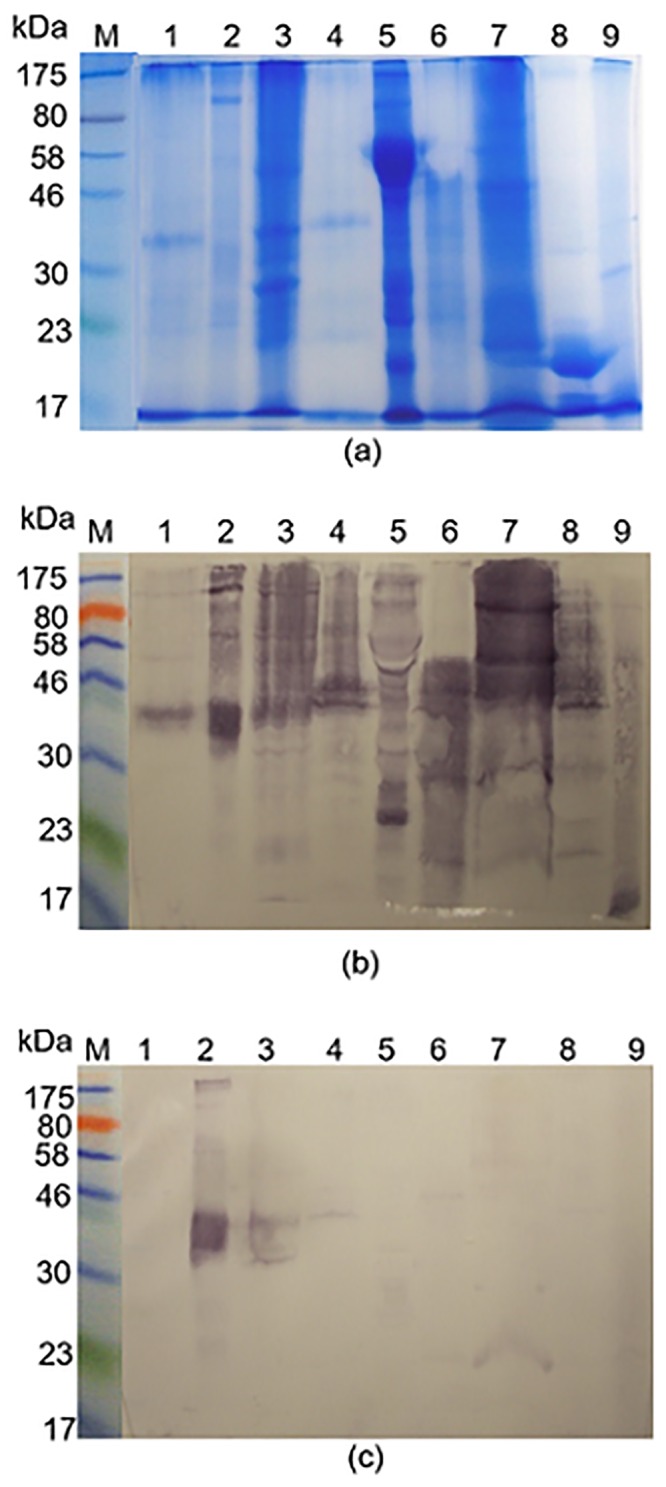
PAGE and electroblots of allergen extracts probed with rabbit anti-SmSEA antiserum. **(a**) Coomassie blue-stained SDS-PAGE gel of allergen extracts and *S*. *mansoni* SEA (SmSEA). **(b)** Western immunoblot of allergen extracts and SmSEA electro-transferred to nitrocellulose film from a replicate of the gel in (a) and probed with a rabbit anti-SmSEA antiserum. **(c)** Western immunoblot replicate of (b) except for treatment of the nitrocellulose film with 10 mM sodium meta-periodate after electro-transfer of allergen/SmSEA molecules and before incubation in primary rabbit anti-SmSEA antiserum. M = molecular size markers; Lanes and (amount of BSA-equivalent protein added to each lane):1 = latex (0.20 mg); 2 = SmSEA (0.010 mg); 3 = banana (0.16 mg); 4 = tomato (0.27 mg); 5 = peanut (0.10 mg); 6 = melon (0.24 mg); 7 = avocado (0.22 mg); 8 = Kiwi fruit (0.19 mg); 9 = chestnut (0.22 mg).

Rabbit antisera raised against SmSEA cross-reacted with electroblotted constituents of latex, banana, tomato, peanut, melon, avocado, kiwi fruit and chestnut (Fig 1b); i.e., a similarly extensive pattern of cross-reactivity as that of anti-Sm480 antibodies in S1 Fig.

### Cross reactivity of rabbit anti-schistosome IgG antibodies with allergens might be due to CCDs

Treatment of membrane-bound allergens with 10 mM sodium meta-periodate resulted in nearly complete loss of the cross-reactivity of the rabbit anti-SmSEA antibodies with the allergens. Notably, reactivity against the schistosome antigens was not affected (Fig 1c, lane 2). Similar results were obtained using the rabbit anti-Sm480 antibody (S3 Fig). These data suggest that the schistosome—allergen cross-reactivity is due to the presence of identical or very similar carbohydrate structures on both schistosome antigens and allergens, so-called cross-reactive carbohydrate determinants (CCDs).

Control experiments were performed to investigate whether sera from rabbits generally contain IgG which reacts ‘non-specifically’. Tests with IgG purified from the sera of two rabbits immunized, respectively, with different *S*. *mansoni* worm antigens, namely aldolase and alkaline phosphatase, indicated they did not have antibodies that reacted with allergen extracts (S4 Fig), nor did serum from a rabbit injected with Freund’s adjuvant alone. The cross-reactivity of the IgG in the anti-SmSEA and anti-SmCH sera was also not completely unrestricted as neither type reacted with electroblotted extracts of normal mouse blood, kidney and spleen (S5 Fig). Most of a large number of other rabbit antisera also did not react against these mouse tissues (S5 Fig).

### A 43 kDa rubber latex molecule that is recognized by rabbit anti-*S*. *mansoni* IgG antibodies

Both, rabbit anti-SmSEA and anti-Sm480 IgG antibodies recognized one prominent band of approximately 43 kDa in the latex extract (Fig 1b, lane 1, and S1 Fig).

**Fig 2:**
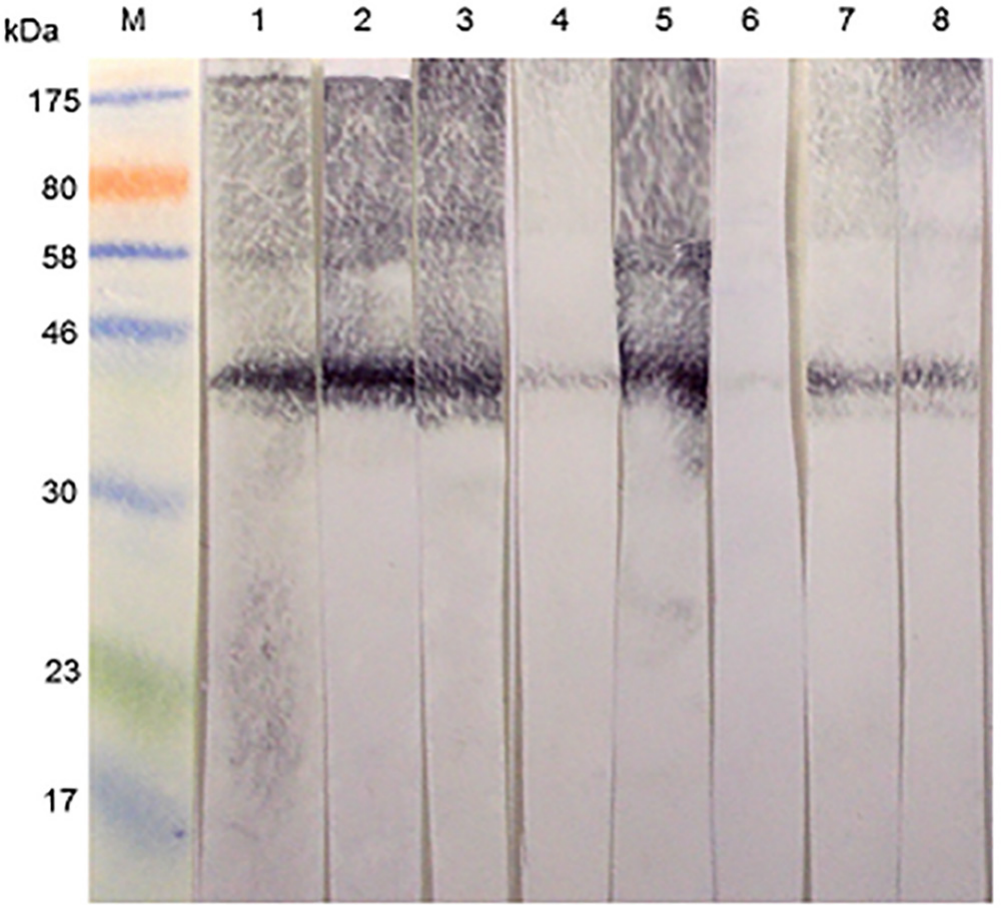
Reactivity of a 43 kDa latex molecule with rabbit antisera raised against *S*. *mansoni* cercarial (SmCH) and egg (SmSEA) antigens. M = molecular size markers. Lanes 1–3 were probed with 3 different rabbit antisera raised against homogenates of *S*. *mansoni* cercariae. Lanes 4–8 were probed with sera from 5 different rabbits immunized with homogenates of *S*. *mansoni* eggs. Amount of protein in latex extract across all the 8 lanes = 1.80 mg. The serum used for lane 5 was the same as that used in Fig 1.

Interestingly, this band was also recognized by rabbit sera raised against *S*. *mansoni* cercarial antigens (SmCH) as shown in Fig 2, lanes 1–3. Moreover, all three investigated anti-SmCH antisera had more intense reactivity than 4 rabbit anti-SmSEA antisera (Fig 2,lanes 4 and 6–8), which were investigated in addition to the one used in the previous experiments (Fig 2, lane 5).

**Fig 3:**
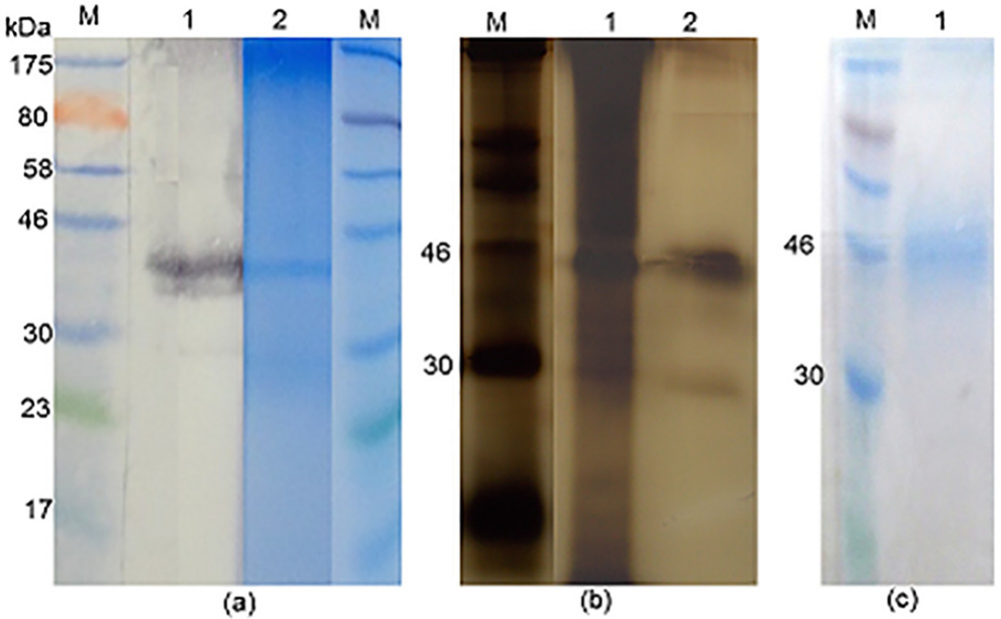
Purification of a 43 kDa latex antigen that is cross-reactive with *S*. *mansoni* for mass spectrometric analysis. **(a)** Two lanes to the left: M = molecular size markers; 1 = Western immunoblot reactivity of a rabbit anti-SmSEA against latex extract. Two lanes to the right: Coomassie blue-stained SDS-PAGE gel: 2 = latex extract; M = molecular size markers. 0.02 mg of protein was added to each latex lane. **(b)** Silver-stained SDS-PAGE gel. M = molecular weight markers; 1 = untreated latex extract; 2 = result of purification of 43 kDa latex molecule by repeated immunoelectrophoreses. **(c)** Coomassie-stained SDS-PAGE gel. M = molecular size markers; 1 = result of purification of 43 kDa latex molecule by a repeated immunoelectrophoresis. Arrow indicates the stained band that was subjected to mass spectrometric analysis.

In order to identify the cross-reactive 43 kDa protein in the latex extract, the respective band was excised from SDS-PAGE gels and subjected to mass spectrometric analysis. Thus, as indicated in Fig 3a, a protein band in a Coomassie blue-stained SDS-PAGE gel is present in a position that corresponds to the immuno-reactive 43 kDa molecule in the western blots. The protein was purified by excising the band from replicate gels, eluting the protein from the gel strips in Tris-HCl buffer, pH 7.4, concentrating the eluate and re-electrophoresing the product. The procedure was repeated once, with the result shown in Fig 3c. Fig 3b is an image of a silver-stained gel showing that the purification procedure had resulted in removal of much of the extraneous background material.

The purified material (arrow in Fig 3c) was subjected to mass spectrometric analysis and the results are given in Table 1. Peptide matches (listed in Table 1) were found for one protein entry in the NCBInr database, namely *Hevea brasiliensis* allergen Hev b 7, which has a calculated predicted mass of 42.8 kDa. Two peptides from Hev b 1, a molecule with a calculated mass of ~14 kDa, were also detected in the MS analysis of the purified protein.

**Table 1:** MASCOT search output of tandem MS data from the purified 43 kDa gel band purified from latex extract.

gi: 3087805, latex allergen Hev b 7 [*Hevea brasiliensis*]		
**Mass:** 42845 **Score:** 201 **Matches:** 8(0) **Sequences:** 5(0)		
**Peptide match**	**Score**	**Expect**
GIIPGIILASLESK	54	1
LQDLDGPDAR	45	11
ATGSTTLTQGKK	42	23
ITVLSIDGGGIR	40	32
LLLPVIFSSDDAK	20	3.5e+03
Percentage sequence coverage: 15%.		
Matched peptides shown underlined.		
1 MATGSTTLTQ GKKITVLSID GGGIRGIIPG IILASLESKL QDLDGPDARI		
51 ADYFDIIAGT STGGLITTML TAPNEDKKPM YQAKDIKDFY LENCPKIFPK		
101 ESRDNYDPIH SIGPIYDGEY LRELCNNLLK DLTVKDTLTD VIIPTFDIKL		
151 LLPVIFSSDD AKCNALKNAR LADVCISTSA APVLLPAHSF TTEDDKNIHT		
201 FELIDGGAAA TNPTLLALTH IRNEIIRQNP RFIGANLTES KSRLVLSLGT		
251 GKSEYKEKYN ADMTSKWRLY NWALYNGNSP AVDIFSNASS DMVDFHLSAL		
301 FKSLDCEDYY LRIQDDTLTG EESSGHIATE ENLQRLVEIG TRLLEKQESR		
351 INLDTGRLES IPGASTNEAA ITKFAKLLSE ERKLRQLK		

### Identity of *S*. *mansoni* antigens which are cross-reactive with the rubber latex allergen Hev b 7

**Fig 4:**
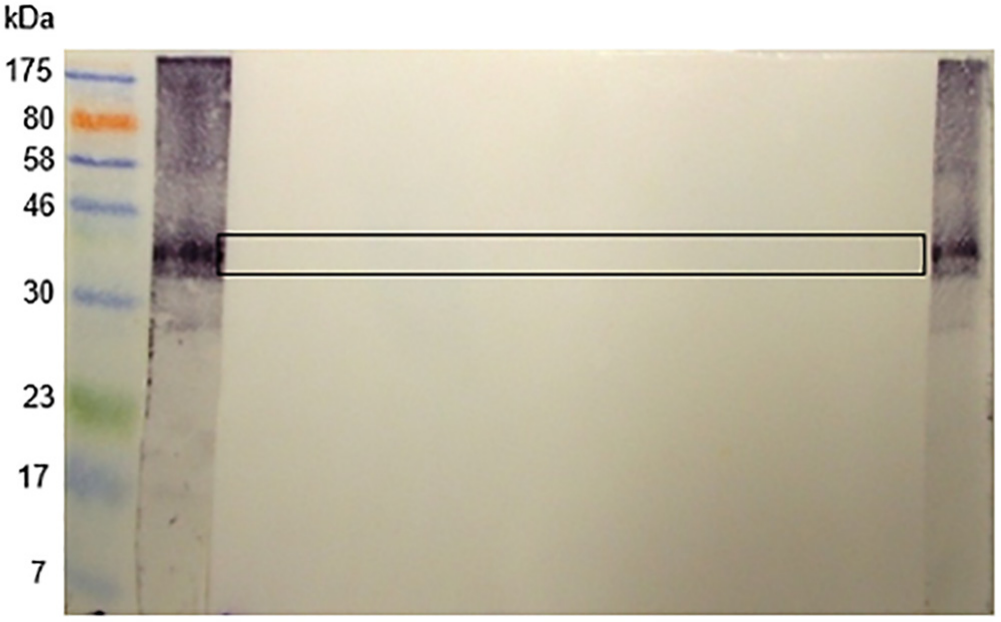
Illustration of protocol used to purify rabbit anti-SmSEA antibodies that are cross-reactive with a 43 kDa latex molecule by acid-elution from electroblot of latex extract. M = molecular size markers. WB = 2 edges of the nitrocellulose film subjected to western immunoblotting procedure to indicate position of 43 kDa latex molecule. Horizontal rectangular outline = area excised from film and used to purify latex cross-reactive, rabbit anti-SmSEA primary antibodies by acid-elution.

In order to identify the latex cross-reactive *S*. *mansoni* egg antigens, we purified the rabbit anti-*S*. *mansoni* egg antibodies specific for the 43 kDa latex allergen Hev b 7, as demonstrated in Fig 4. The area of the NCP outlined by the horizontally-marked rectangle, which the result obtained after completion of the immunoblotting on the 2 edge strips indicates was carrying immune complexes of rabbit antibody and the 43 kDa molecule, was excised and the strip incubated momentarily in low pH buffer to elute the primary antibody. The acid-eluted antibodies were used to probe electroblotted extracts of schistosome eggs (SmSEA), cercariae (SmCH) or adults (SmWH) or the latex extract containing the 43 kDa molecule, putatively identified as Hev b 7, as a control.

**Fig 5:**
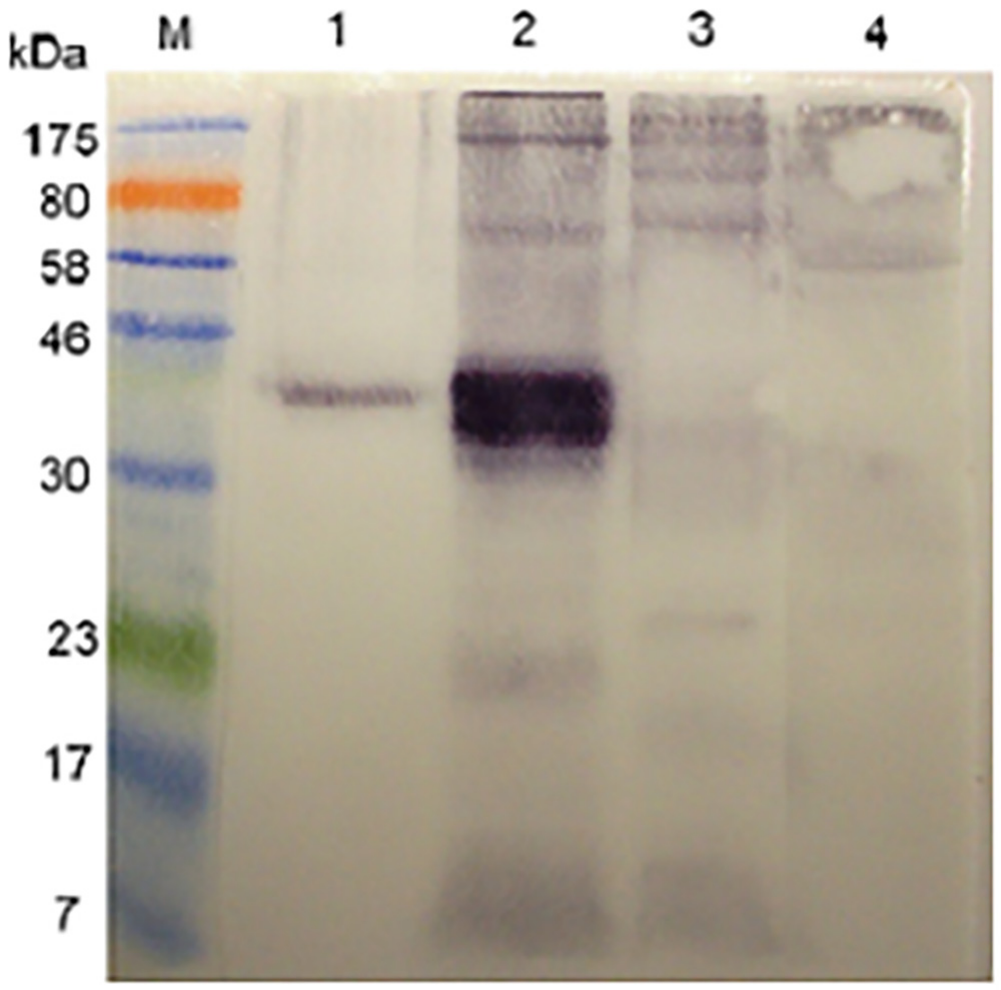
Western blot showing reactivity of acid-eluted, latex cross-reactive, rabbit anti-SmSEA antibodies against latex extract and extracts of *S*. *mansoni* eggs, cercariae and worms. M = molecular size markers. Antigens in lanes: 1 = latex extract; 2 = SmSEA; 3 = SmCH (0.025 mg); 4 = SmWH (0.025 mg).

As expected, the eluted antibodies reacted against a 43 kDa band in the latex extract (Fig 5, lane 1). They also reacted intensely with a pair of bands of ~40 kDa in SmSEA, somewhat less intensely with a narrow band at the upper end of the SmSEA lane and only smearingly and lightly with other constituents of SmSEA (Fig 5, lane 2). There was only some low-intensity, smear-like activity against the constituents of SmCH and SmWH (Fig 5, lanes 3 and 4).

**Fig 6:**
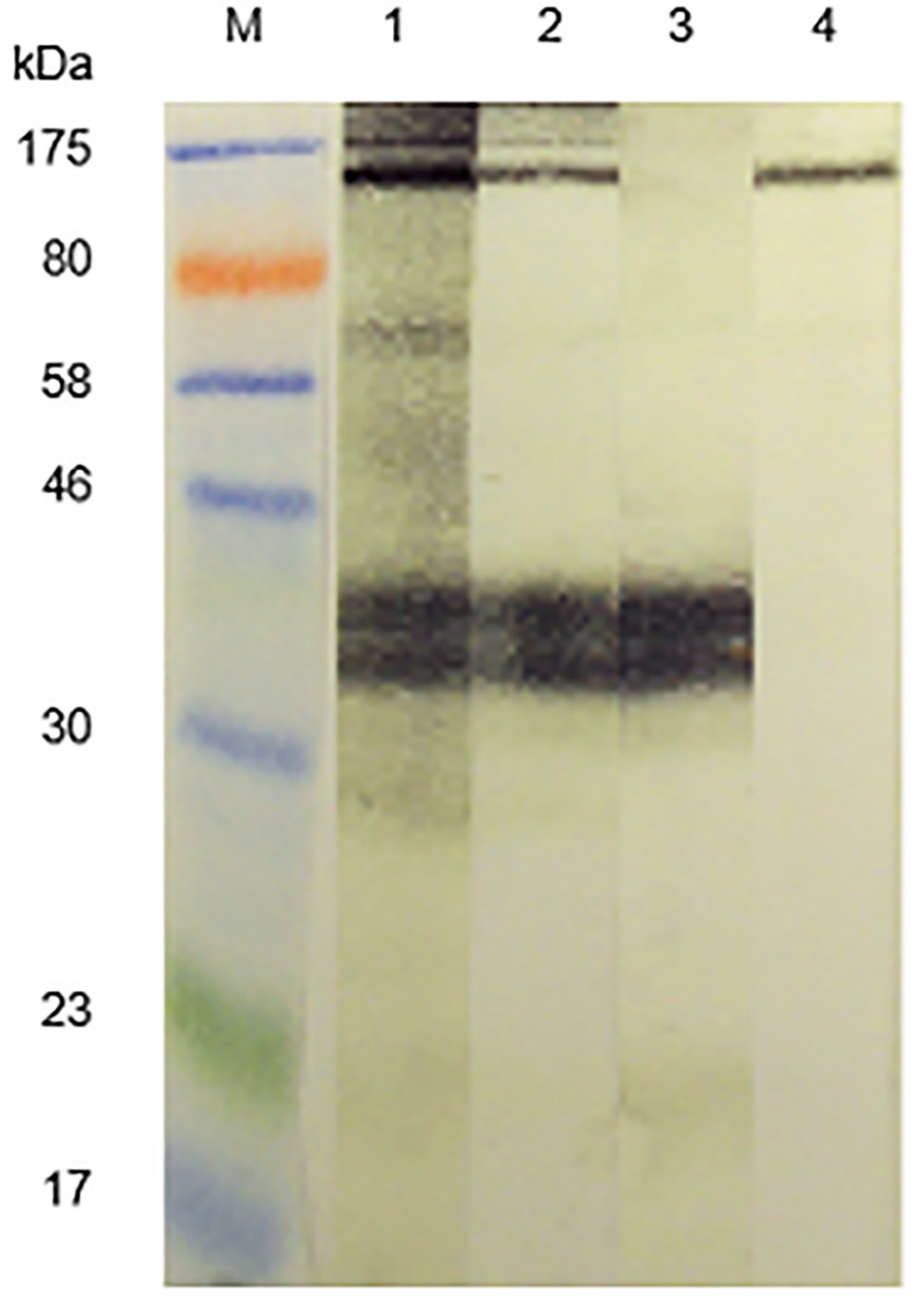
Western blot immunoreactivity of acid-eluted antibodies and control rabbit antisera monospecific for *S*. *mansoni* egg antigens; IPSE/alpha-1 and kappa-5 against *S*. *manson*i egg antigens. M = molecular size markers. Primary antibodies: 1 = anti-SmSEA; 2 = anti-SmSEA antibodies eluted from the 43 kDa latex molecule; 3 = rabbit antiserum specific for *S*. *mansoni* egg antigen IPSE/alpha-1; 4 = rabbit antiserum specific for *S*. *mansoni* egg antigen kappa-5.

It was suspected that the pair of ~40 kDa SmSEA antigens and the higher molecular weight SmSEA molecules reacting with the eluted molecules could have been, respectively, IPSE/alpha-1 [66] and kappa-5 [67]. To test this hypothesis the immunoblot pattern given by the acid-eluted rabbit antibodies (as had been used in Fig 5) was compared with that given by rabbit antisera that were, respectively, specific for IPSE/alpha-1 and kappa-5. As shown in Fig 6, the two monospecific rabbit antisera did react against a ~40 kDa pair of proteins (i.e., IPSE/alpha-1) and the larger molecule (kappa-5) in SmSEA in a similar manner to the anti-SmSEA antibodies that had been eluted from the 43 kDa latex molecule.

### Identity of S. *mansoni* cercarial antigens that are cross-reactive with Hev b 7

**Fig 7:**
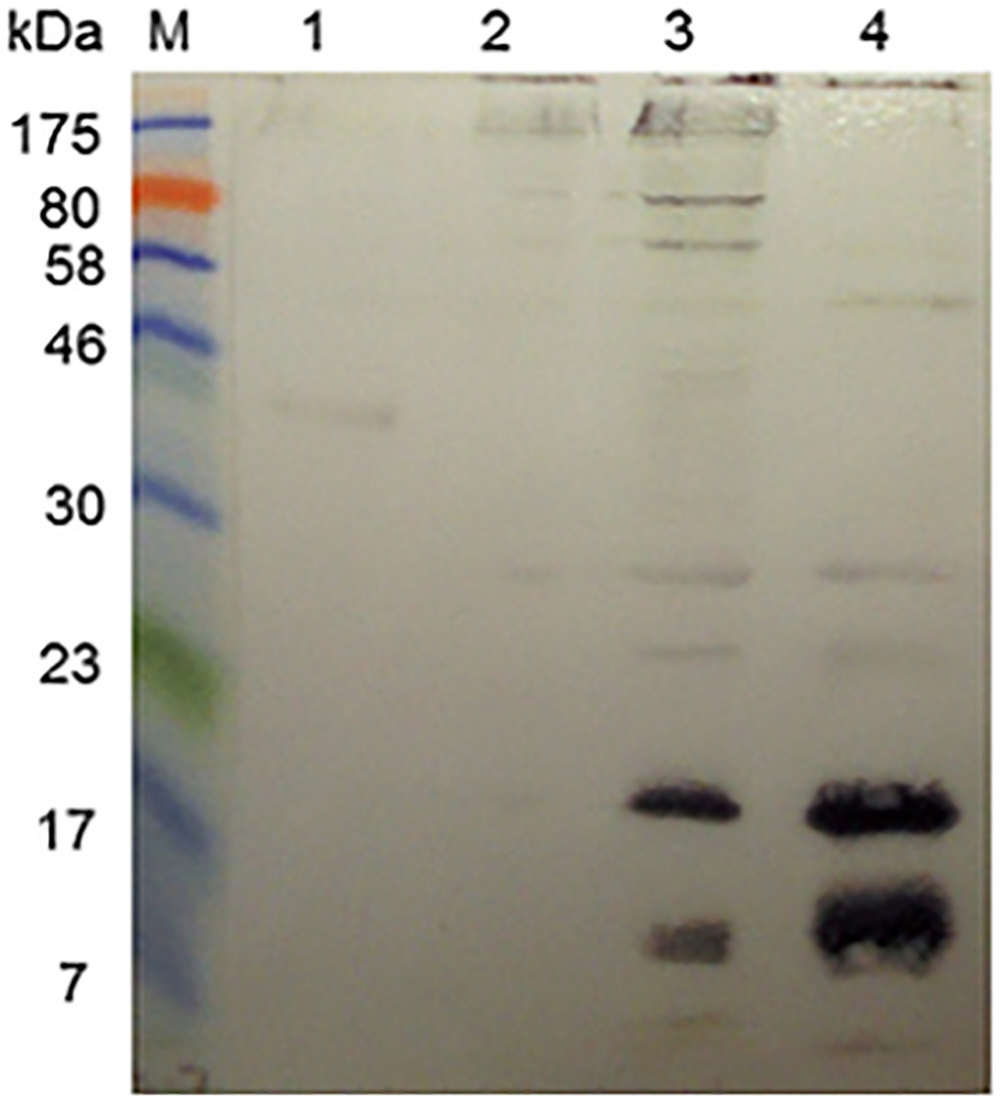
Reactivity of acid-eluted anti-*S*. *mansoni* cercarial antibodies that cross-reacted with the 43 kDa latex molecule. M = molecular size markers. Antigens in lanes: 1 = latex extract; 2 = SmSEA; 3 = SmCH; 4 = SmCTF (0.025 mg). Immunoblot was probed with rabbit anti-SmCH antibodies purified by acid-elution from 43 kDa latex molecule.

In Fig 2 it was shown that antibodies in 3 rabbit antisera raised against SmCH also reacted against the 43 kDa latex molecule, as did the rabbit anti-SmSEA serum that had been used for Fig 1. Similar experiments to those described above for the anti-SmSEA, latex cross-reactive antibodies in Figs 4 and 5 were therefore undertaken to try and find out which *S*. *mansoni* cercarial antigens may have induced the antibodies that cross-reacted with the latex molecule. Rabbit anti-SmCH antibodies that had reacted with the latex molecule were thus acid-eluted as for the latex-reactive anti-SmSEA antibodies, concentrated, pooled and tested for their reactivity against *S*. *mansoni* antigens in SmSEA, SmCH and SmCTF, as well as the latex extract as a control. The results are given in Fig 7. Two relatively low molecular weight constituents (20 kDa and 14 kDa) in both the cercarial antigen preparations reacted with the eluted antibodies (Fig 7, lanes 3 and 4), with some less intense reactivity against other molecules in these extracts. There was very little reactivity against SmSEA (Fig 7, lane 2) and some barely visible reactivity against the 43 kDa molecule in the latex extract (Fig 7, lane 1).

**Fig 8:**
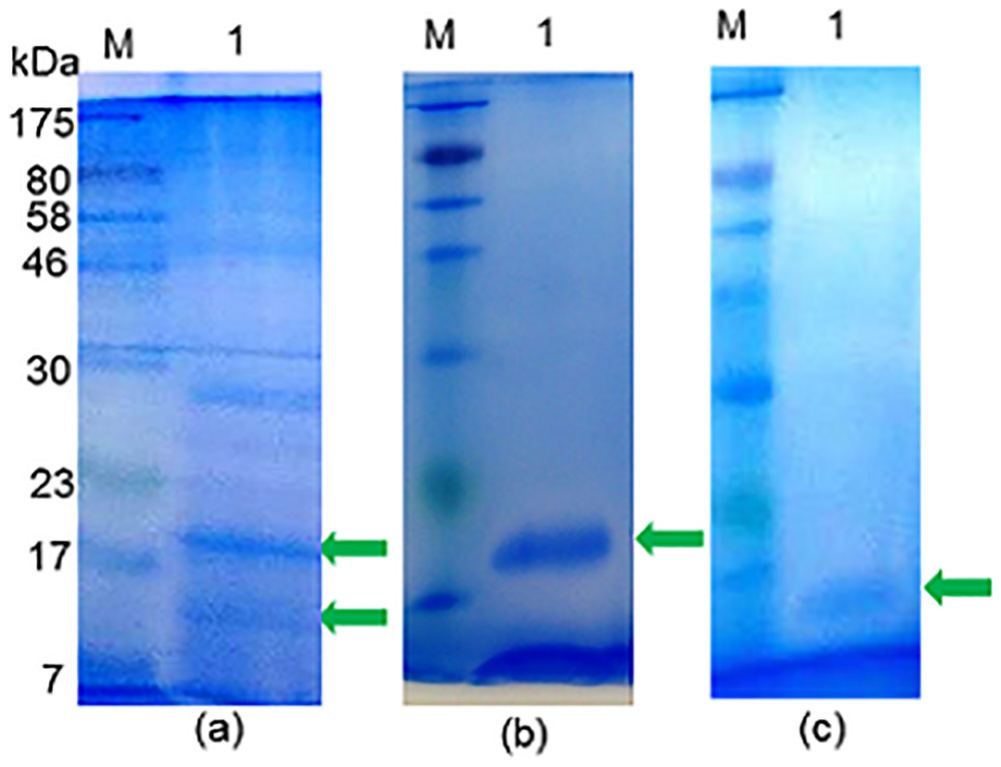
Purification of the two *S*. *mansoni* cercarial antigens which reacted with the acid-eluted antibody in Fig 7. **(a)** Coomassie blue-stained SDS-PAGE of SmCTF. Arrows indicate the two protein bands of 20 kDa and 14 kDa, which were excised, eluted from the gel and re-electrophoresed in order to purify for mass spectrometric analysis. M = molecular size markers; 1 = lane loaded with SmCTF. **(b)** Coomassie blue-stained SDS-PAGE gel. Arrow indicates purified 20 kDa protein from SmCTF in lane 1. **(c)** Coomassie blue-stained SDS-PAGE gel. Arrow indicates purified 14 kDa protein in lane 1.

In order to further characterize the two *S*. *mansoni* cercarial antigens that had ostensibly induced the rabbit antibodies that cross-reacted with the latex molecule the former were purified by repeated resolutions in SDS-PAGE gels and elutions, followed by mass spectrometric analysis. The outcome of the purification procedure is illustrated in Fig 8, panel ‘b’ for the 20 kDa molecule and ‘c’ for the 14 kDa molecule and the two protein samples were subjected to mass spectrometric analysis. The results for the 20 kDa and 14 kDa molecules are given in Tables 2 and 3 respectively. Significant matches for the peptides derived from the 20 kDa SmCH molecule identified by MS and listed in Table 2 were found in one protein entry in the NCBInr database, namely *S*. *mansoni* protein SPO-1, a molecule with a calculated predicted mass of 13.6 kDa. Peptides from the 14 kDa molecule were found to match Sm14 (SwissProt database), a fatty acid-binding protein with a predicted mass 14.8 kDa (Table 3).

**Table 2:** MASCOT search output of tandem MS data from the purified ~20 kDa gel band from *S*. *mansoni* cercariae.

gi: 4588483, SPO-1 protein [*Schistosoma mansoni*]		
**Mass:** 13642 **Score:** 175 **Matches:** 7 (1) **Sequences:** 3 (1)		
**Peptide match**	**Score**	**Expect**
*DMELVYIDAEYEK*	71	0.03
TTLEQAPHPSEKDMELVYIDAEYEK	59	0.74
TTLEQAPHPSEK	52	2.3
Percentage sequence coverage: 21%.		
Matched peptides shown underlined.		
1 MKVTPIIFAV FCVVGAMTLI TATTLEQAPH PSEKDMELVY IDAEYEKEGG		
51 LKSICNEIKR SFRKGRHHIY KVMDKYIRKE DLGMKMLDVA KILGRRIEKR		
101 MEYIAKKLDK MMEYESS		

**Table 3:** MASCOT search output of tandem MS data from the purified ~14 kDa gel band from cercariae.

FABP_SCHMA, 14 kDa fatty acid-binding protein OS = *Schistosoma mansoni* PE = 1 SV = 1,		
**Mass:** 14838 **Score:** 82 **Matches:** 3 (0) **Sequences:** 3 (0)		
**Peptide match**	**Score**	**Expect**
TTVTVGDVTAIR	34	1.3
FGEEFDEK	26	9.0
LTQTQVDPK	23	18.0
Percentage sequence coverage: 21%.		
Matched peptides shown underlined.		
1 MSSFLGKWKL SESHNFDAVM SKLGVSWATR QIGNTVTPTV TFTMDGDKMT		
51 MLTESTFKNL SCTFKFGEEF DEKTSDGRNV KSVVEKNSES KLTQTQVDPK		
101 NTTVIVREVD GDTMKTTVTV GDVTAIRNYK RLS		

### Reactivity of schistosome/latex cross-reactive antibodies with other allergens

Human subjects suffering from allergy to latex are commonly known to be also allergic to a variety of food allergens, the so-called latex-fruit syndrome and it is possible that the syndrome is due to antigenic cross-reactivity between latex and the food allergens [45, 46, 68]. The possibility that the rabbit anti-SmSEA antibodies that had been eluted from the 43 kDa latex molecule cross-reacted with constituents of extracts of fruits implicated in the latex-fruit syndrome was therefore investigated.

**Fig 9:**
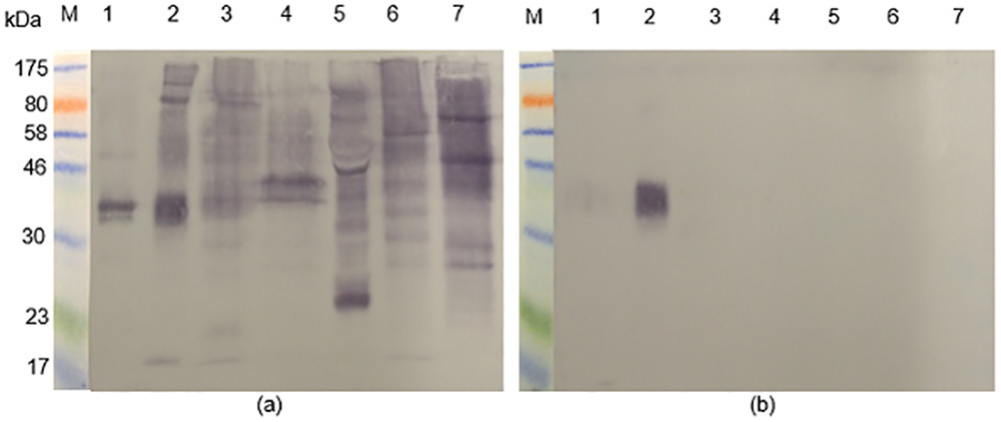
Reactivity of acid-eluted anti-SmSEA-derived anti-latex antibodies against extracts of some allergens known to be implicated in the latex-fruit syndrome. **(a)** Western immunoblot of allergen extracts and SmSEA electrophoreses probed with rabbit anti-SmSEA antibodies that were cross-reactive with a 43 kDa latex molecule. **(b)** Western immunoblot replicate of (a) except for treatment of the nitrocellulose film with 20 mM sodium meta-periodate after electro-transfer of allergen/SmSEA molecules and before incubation in primary rabbit anti-SmSEA antiserum. M = molecular size markers; 1 = latex; 2 = SmSEA; 3 = banana; 4 = tomato; 5 = peanut; 6 = melon; 7 = avocado. The amount of protein in each lane (BSA-equivalent) is the same as in Fig 1.

Fig 9a shows that the eluted antibodies did indeed react with extracts of several foods, including banana, tomato, peanut, melon and avocado, all of which are often found in lists of allergens implicated in the latex-fruit syndrome.

Treatment of the NCP on to which the above-mentioned had been electroblotted with 20 mM sodium meta-periodate prior to application of the eluted antibodies abolished the antigenic cross-reactivity in all six allergen extracts (Fig 9b). Once again, the antigenic reactivity of a ~40 kDa pair of molecules in SmSEA (Fig 9b, lane 2) was not affected by the periodate treatment (compare with Fig 1c, lane 2). This result is consistent with involvement of CCDs in the antigenic cross-reactivity between *S*. *mansoni* antigens and allergens known to be involved in the latex fruit syndrome.

## Discussion

Initial preliminary experiments showed that antibodies in a rabbit serum with specificity for Sm480, a glycosylated *S*. *mansoni* egg antigen [33] were extensively antigenically cross-reactive with the constituents of a wide variety of plants and invertebrates known to be allergic in humans (S1 Fig). In this extension of the previous work rabbit antisera raised against other unfractionated *S*. *mansoni* egg and cercarial homogenates were tested and also showed cross-reactivity against aqueous extracts of allergens (Figs 1 and 2).

Results of control experiments to investigate whether sera from rabbits generally contain IgG which react ‘non-specifically’ indicated for the most part they did not (S4 and S5 Figs).

Of the aqueous extracts from plants which showed cross-reactivity with rabbit anti-Sm480 and rabbit anti-SmSEA and anti-SmCH sera, one prepared from a commercially-available rubber latex-based adhesive product (Copydex) was somewhat distinct in containing relatively few antigenically cross-reactive protein bands, one of which was approximately 43 kDa. We chose this particular system of antigenic cross-reactivity for further investigation because of its relative simplicity.

The 43 kDa latex-derived molecule was purified and subjected to mass spectrometry and identified as Hev b 7. Hev b 7 is a designated allergen in natural rubber latex and belongs to the patatin gene family [69–71]. Investigations using western immunoblotting have revealed that 11% of individuals allergic to natural latex expressed IgE antibodies to both recombinant and natural forms of Hev b 7 [71].

*S*. *mansoni* egg and cercarial antigens with which Hev b 7 was here found antigenically cross-reactive have been also tentatively identified. Thus, rabbit anti-*S*. *mansoni* egg (SmSEA) IgG antibodies that were purified by acid-elution from electroblotted Hev b 7 reacted with a molecule of ~100 kDa and more conspicuously with a 40 kDa pair of molecules in SmSEA (Fig 5). By virtue of the similarity of reactivity of the purified antibodies with the respective reactivities of rabbit monospecific antisera we consider the two *S*. *mansoni* egg molecules most likely to be, respectively, IPSE/alpha-1 [66] and kappa-5 [67].

IPSE/alpha-1 is an IgE-binding protein capable of inducing the release of interleukin (IL)-4 from basophils [72] and its amino acid sequence includes a nuclear localization signal [73]. However, neither of these biological properties of IPSE/alpha-1 seems likely to be relevant to the antigenic cross-reactivity investigated here.

The molecule does also to some extent bind IgG in a ‘non-specific’ manner (i.e., by a mechanism that does not involve the immunoglobulin’s antigen-binding site), but with a 4-fold lower affinity than to IgE [72]. Fig 6, lane 4 and Fig 7, lane 2 provide some evidence that in the present experiments rabbit IgG did not react ‘non-immunologically’ with IPSE/alpha-1. Thus, in Fig 5 the rabbit anti-kappa-5 antiserum reacted only with a much larger molecule than IPSE/alpha-1 and in Fig 7 the anti-SmCH IgG that was purified by acid-elution from electroblotted cercarial antigens did not react with any constituents in SmSEA. Furthermore, in S4 Fig the rabbit anti-Sm AKP and rabbit anti-Sm aldolase antisera did not react with any ~40 kDa molecule in SmSEA.

No biological function has so far been attributed to *S*. *mansoni* egg antigen kappa-5.

Purified rabbit anti-*S*. *mansoni* cercarial (SmCH) IgG antibodies were reactive with two cercarial antigens (Fig 7) which were purified (Fig 8) and which mass spectrometric analysis indicated were, respectively, 16 kDa *S*. *mansoni* Sm16/SPO-1 (GI: 8468613, AAF75550.1|AF269252_1) and a 14 kDa fatty acid binding protein (FAB) (FABP SCHMA: P29498). Sm16/SPO-1 has been implicated in inducing the anti-inflammatory cytokine, IL-10 and thus involved in host immunomodulation [74], while, as its name suggests, FABP/Sm14 plays an important role in the binding and transport of lipids and is being developed as a schistosomiasis vaccine candidate [75, 76].

The question arises as to why *S*. *mansoni* egg and cercarial antigens are antigenically cross-reactive with the constituents of extracts of many different allergens, and here with rubber latex molecule Hev b 7 in particular? Alignment of the amino acid sequences of each of the four *S*. *mansoni* antigens with that of Hev b 7 in every case gave a non-significant level of similarity between them and thus the linear amino acid sequences themselves may not explain the antigenic cross-reactivity demonstrated here. As already mentioned, Hev b 7 is a member of the patatin family of proteins, yet there is no evidence to suggest that *S*. *mansoni* molecules IPSE/alpha-1, kappa-5, Sm16/SPO-1 or Sm14/FABP are at all patatin-like, the recent suggestion that there is significant similarity, inclusive of the epitopic regions, between allergens and helminth proteins [12, 13] notwithstanding.

The antigenic cross-reactivities studied here may however be due to the presence of identical glycanic epitopes (cross-reactive carbohydrate determinants: CCDs) on many different molecules in the schistosome and allergen extracts. CCDs, especially those that are N-linked, have been shown to be important in the cross-reactivity between plants and some invertebrates and structures such alpha 1,3 fucose attached to a proximal N-acetyl glucosamine and beta 1,2 xylose attached to a core mannose have been implicated [77, 78].

As illustrated in S3 Fig, virtually all the reactivity of rabbit anti-Sm480 IgG antibodies against antigens in extracts of cockroach, birch pollen, house dust mite, avocado and bee venom was abolished by treatment with sodium meta-periodate, an oxidative reaction that destroys hexose and pentose structures. A similar result was obtained with the anti-SmSEA antibodies (Fig 1b). Similarly, sodium meta-periodate-treatment destroyed the reactivity of the anti-SmSEA antibodies that had been eluted from the 43 kDa latex antigen against molecules in a variety of allergen extracts (Fig 9). Note that a concentration of 20 mM sodium meta-periodate was used in the experiment in Fig 9, resulting in a somewhat greater clearance of antigenic reactivity in the allergen lanes, particularly lane 3 (banana) than in Fig 1c, in which experiment 10 mM sodium meta-periodate was used. These results are consistent with the cross-reactivity between *S*. *mansoni* and latex and other allergens being due to CCDs. The variation in anti-latex reactivity of the 5 rabbit anti-SmSEA antisera in Fig 2 may be due to differences between the rabbits with respect to their anti-CCD responses.

The results in Figs 5 and 6 suggest that the *S*. *mansoni* egg antigens that induced latex cross-reactive antibodies were IPSE/alpha-1 and kappa-5. IPSE/alpha-1 is a dimeric glycoprotein and each of its peptides has two potential sites for N-linked glycosylation. To one or both of these may be attached a di-antennnary core region that is di-fucosylated and one or two Lewis X antennae which are immunogenic [79]. Xylose was however not detected in the glycanic residues of IPSE/alpha-1 [79]. Kappa-5 [67] is likewise glycosylated: it has four N-linked glycosylation sites bearing unique tri-antennary structures consisting of a core region of both fucose and xylose and an immunogenic terminal composed of LDN (GalNacβ1-4GlcNAc) [80].

Paradoxically, periodate-treatment did not abolish the reactivity of the eluted antibodies against a pair of 40 kDa proteins in SmSEA, putatively IPSE/alpha-1. A capacity of rabbit antibodies to bind to this egg antigen after periodate-treatment, but not to other similarly-treated *S*. *mansoni* egg antigens, has been noted before [58]. As argued above, it seems unlikely that the cross-reactivity between IPSE/alpha-1 and Hev b 7 is due to homologous amino acid sequence(s). Hino et al., found that some mice immunized with horse radish peroxidase produced anti-CCD antibodies that cross-reacted with phytohaemagglutinin even after periodate treatment of the latter and they suggested that the antibodies in these mice recognized the trisaccharide backbone of the CCD, rather than the xylose, fucose and mannose residues that would be most likely subject to degradation by the periodate [81]. However, the reason why IPSE/alpha-1 retains its antigenicity after periodate-treatment, while allergen molecules that are antigenically cross-reactive with IPSE/alpha-1 have lost theirs, remains to be determined.

Although no N-linked glycosylation site was found in the amino acid sequence of *S*. *mansoni* Sm16/SPO-1, there are two sites potentially allowing O-linked glycosylation. Conversely, *S*. *mansoni* Sm14/FABP has two potential N-linked and fifteen O-linked glycosylation sites. It is worth noting that the O-glycans in plants and invertebrates have been only poorly investigated so far [82], though O-glycan-borne epitopes on Art v 1, an allergen in mugwort pollen, are antigenic in immunoblots with IgE antibodies in sera from mugwort allergic patients and with rabbit IgG antibodies [83]. O-linked glycans thus may play a role in the antigenic cross-reactivity between *S*. *mansoni* cercarial antigens and Hev b 7.

Software-based analysis of the amino acid sequence of Hev b 7 with GlycoEP suggests three N-linked and twenty-nine O-linked glycosylation sites. However, Sowka et al., reported that Hev b 7 is not glycosylated [70]. We have no explanation that may help resolve this potentially conflicting observation, made additionally complicated by virtue of this molecule seemingly carrying epitopes that are separately specifically cross-reactive with either *S*. *mansoni* egg antigens or cercarial antigens (Figs 5 and 7). Further investigations are therefore needed critically to analyse the structures of this latex molecule and the schistosome antigens implicated in this cross-reactivity and elucidate which epitopes are involved in the IgG cross-reactive reactions observed here.

In summary, the present study has used rabbit antisera to demonstrate antigenic cross-reactivity between *S*. *mansoni* antigens and the constituents of a variety of allergens, with reactivity of rabbit anti-schistosome IgG antibodies against a molecule of 43 kDa in a rubber latex extract having been studied in particular. Mass spectrometry identified the 43 kDa schistosome cross-reactive latex molecule as allergen Hev b 7. Rabbit anti-schistosome IgG antibodies eluted from electroblotted Hev b 7 in turn reacted with *S*. *mansoni* egg antigens IPSE/alpha-1 and kappa-5, and *S*. *mansoni* cercarial antigens Sm16/SPO-1 and a fatty acid-binding protein Sm14/FABP. These eluted antibodies also cross-reacted with antigenic constituents of a variety of allergens, a result of potential relevance to the latex-fruit syndrome of allergic reaction [45–47]. Most of this cross-reactivity was destroyed by treatment of the electroblotted antigens with sodium meta-periodate, a result again consistent with the cross-reactivity being due to CCDs. The same applies to the cross-reactivity found between latex and hymenoptera venoms [84].

Natural rubber latex has, however, now been largely replaced with other less allergenic materials, so rates of allergic sensitization to latex have decreased [85] compared with former times when it affected a significant number of those who came into contact with it, particularly health-care workers [86] and spina bifida patients [87]. (Incidentally, Hev b 7 has been directly implicated in latex allergy in spina bifida children [88]).

Using the present study as a template, work similar to that described here is currently underway investigating in more detail patterns of cross-reactivity between *S*. *mansoni*-induced rabbit IgG antibodies and other, perhaps now more commonly encountered allergens, such as peanut, grass and tree pollens and house dust mite (cross-reactions of which with *S*. *mansoni* are illustrated in S1 Fig and Fig 1). We have also begun to investigate whether sera from schistosome-infected human subjects contain IgG antibodies that cross-react with common allergens. Interestingly, authors of a recent study on human allergy to peanuts in West Africa concluded that: ‘… parasite- (including *S*. *haematobium*-) induced IgE against CCDs might account largely for high IgE levels to peanut in (their) study population of Ghanaian schoolchildren …’, though no evidence of IgE-mediated peanut allergy was found [89]. With regard to the study here it is worth noting that although kappa-5 has been reported to be a target of some IgE antibodies in sera from *S*. *mansoni*-infected individuals [80], none of the 4 schistosome antigens that were cross-reactive with Hev b 7 has been demonstrated to be pathologically allergenic. Is the absence of peanut allergy in the aforementioned Ghanaian schoolchildren study a consequence of the presence of parasite- (schistosome-) induced peanut-cross-reactive IgG antibodies, and might antigens such as *S*. *mansoni* IPSE/alpha-1 also induce such blocking IgG antibodies?

## Supporting Information

S1 Fig Western blot showing reactivity of rabbit anti-Sm480 antibodies against extracts of different foods, plants and invertebrates. Amount of protein applied to each lane is given in brackets. 1 = bee venom (0.020 mg); 2 = peanut (0.011 mg); 3 = banana (0.010 mg); 4 = strawberry (0.004 mg); 5 = melon (0.005 mg); 6 = avocado (0.007 mg); 7 = tomato (0.006 mg); 8 = mushroom 0.007 mg); 9 = birch tree pollen (1.250 mg); 10 = ragweed pollen (0.063 mg); 11 = house dust mite (0.835 mg); 12 = cockroach (0.083 mg); 13 = prawn (0.007 mg); 14 = rubber latex (0.348 mg). MW = molecular size standards (kDa).
(TIF)

S2 Fig Western immunoblot of electroblotted SmSEA probed with antisera from allergen- and Sm480-immunized rabbits. Rabbits were immunized with: 1, bee venom; 2, birch pollen; 3, *S*. *mansoni* egg antigen Sm480. Lane 4 = serum from a rabbit injected with Freund’s adjuvant alone. MW = molecular size standards (kDa). SmSEA load in each lane = 0.010 mg.
(TIF)

S3 Fig Immunoblots of extracts of different allergen extracts probed with rabbit anti-Sm480 antibodies after treatment with sodium meta-periodate. Lanes 1–5: controls treated with sodium acetate buffer alone; lanes 6–10: after treatment with 10 mM sodium meta-periodate in sodium acetate buffer. Lanes 1 & 6 = cockroach; 2 & 7 = birch pollen; 3 & 8 = house dust mite; 4 & 9 = avocado; 5 & 10 = bee venom. Amounts of protein as in S1 Fig.
(TIF)

S4 Fig Immunoblot reactivity of control rabbit antisera against allergens. Purified IgG antibodies from rabbits immunized with **(a)** *S*. *mansoni* aldolase, **(b)** *S*. *mansoni* alkaline phosphatase, and **(c)** serum from a rabbit injected with complete Freund’s adjuvant, reacting against: 1 = SmSEA; 2 = SmWH; 3 = SmCH; and extracts of 4 = natural rubber latex; 5 = peanut; 6 = banana; 7 = tomato; 8 = melon; and 9 = avocado. The amount of protein in each lane (BSA-equivalent) is the same as in main text Fig 1.
(TIF)

S5 Fig Immunoblot reactivity of different rabbit antisera against mouse tissues. Western immunoblots of **(a)** normal mouse serum (NMS), **(b)** an extract of normal mouse kidney, and **(c)** an extract of normal mouse spleen, probed with different rabbit antisera. NMS: 0.003 mg protein (BSA equivalent) was loaded per lane; kidney: 0.021 mg was loaded per lane; spleen: 0.214 mg was loaded per lane. 100 ul of each mixture was loaded into broad wells, non-reduced and not boiled. M = Molecular weight marker; Lanes 1–5 probed with 5 different rabbit anti-SmSEA antisera; 6 & 7 = two anti-SmCH sera; 8 = a rabbit anti-NMS; 9 = a rabbit anti-complete Freund’s adjuvant serum; 10 = rabbit anti-*S*. *mansoni* alkaline phosphatase; 11 = rabbit anti-*S*. *mansoni* aldolase; 12 = rabbit anti-*S*. *mansoni* aldolase; 12 = rabbit anti-*S*. *mansoni* glutathione S-transferase; 13 = anti-*S*. *mansoni* SEA antibodies that had been eluted from the cross-reactive 43 Da latex antigen; 14 = control lane without primary antibody.
(TIF)
