# Supplementary figures and images for: Cross-Reactivity between Schistosoma mansoni Antigens and the Latex Allergen Hev b 7: Putative Implication of Cross-Reactive Carbohydrate Determinants (CCDs)
Source: PLoS One. 2016 Jul 28;11(7):e0159542. doi: 10.1371/journal.pone.0159542 (PMC4965158; doi:10.1371/journal.pone.0159542)

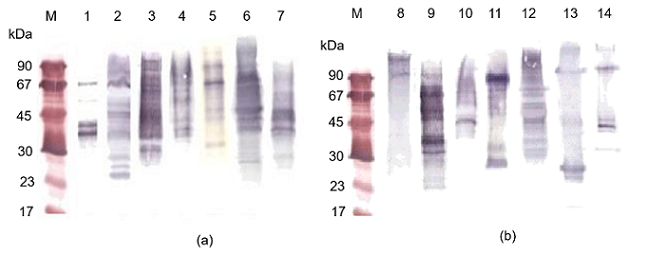

Supplement: S1 Fig — Amount of protein applied to each lane is given in brackets. 1 = bee venom (0.020 mg); 2 = peanut (0.011 mg); 3 = banana (0.010 mg); 4 = strawberry (0.004 mg); 5 = melon (0.005 mg); 6 = avocado (0.007 mg); 7 = tomato (0.006 mg); 8 = mushroom 0.007 mg); 9 = birch tree pollen (1.250 mg); 10 = ragweed pollen (0.063 mg); 11 = house dust mite (0.835 mg); 12 = cockroach (0.083 mg); 13 = prawn (0.007 mg); 14 = rubber latex (0.348 mg). MW = molecular size standards (kDa). (TIF) [file pone.0159542.s001.tif]

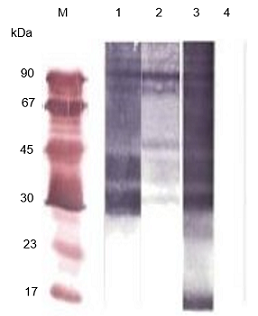

Supplement: S2 Fig — Rabbits were immunized with: 1, bee venom; 2, birch pollen; 3, S. mansoni egg antigen Sm480. Lane 4 = serum from a rabbit injected with Freund’s adjuvant alone. MW = molecular size standards (kDa). SmSEA load in each lane = 0.010 mg. (TIF) [file pone.0159542.s002.tif]

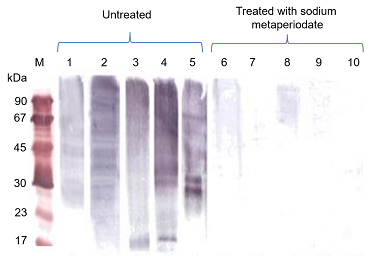

Supplement: S3 Fig — Lanes 1–5: controls treated with sodium acetate buffer alone; lanes 6–10: after treatment with 10 mM sodium meta-periodate in sodium acetate buffer. Lanes 1 & 6 = cockroach; 2 & 7 = birch pollen; 3 & 8 = house dust mite; 4 & 9 = avocado; 5 & 10 = bee venom. Amounts of protein as in S1 Fig. (TIF) [file pone.0159542.s003.tif]

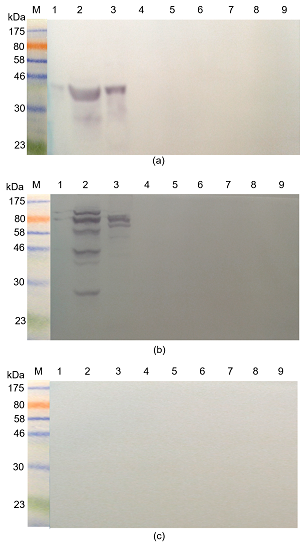

Supplement: S4 Fig — Purified IgG antibodies from rabbits immunized with (a) S. mansoni aldolase, (b) S. mansoni alkaline phosphatase, and (c) serum from a rabbit injected with complete Freund’s adjuvant, reacting against: 1 = SmSEA; 2 = SmWH; 3 = SmCH; and extracts of 4 = natural rubber latex; 5 = peanut; 6 = banana; 7 = tomato; 8 = melon; and 9 = avocado. The amount of protein in each lane (BSA-equivalent) is the same as in main text Fig 1. (TIF) [file pone.0159542.s004.tif]

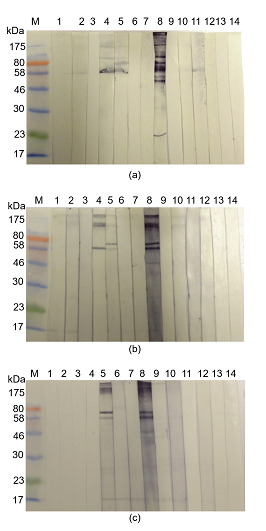

Supplement: S5 Fig — Western immunoblots of (a) normal mouse serum (NMS), (b) an extract of normal mouse kidney, and (c) an extract of normal mouse spleen, probed with different rabbit antisera. NMS: 0.003 mg protein (BSA equivalent) was loaded per lane; kidney: 0.021 mg was loaded per lane; spleen: 0.214 mg was loaded per lane. 100 ul of each mixture was loaded into broad wells, non-reduced and not boiled. M = Molecular weight marker; Lanes 1–5 probed with 5 different rabbit anti-SmSEA antisera; 6 & 7 = two anti-SmCH sera; 8 = a rabbit anti-NMS; 9 = a rabbit anti-complete Freund’s adjuvant serum; 10 = rabbit anti-S. mansoni alkaline phosphatase; 11 = rabbit anti-S. mansoni aldolase; 12 = rabbit anti-S. mansoni aldolase; 12 = rabbit anti-S. mansoni glutathione S-transferase; 13 = anti-S. mansoni SEA antibodies that had been eluted from the cross-reactive 43 Da latex antigen; 14 = control lane without primary antibody. (TIF) [file pone.0159542.s005.tif]
